# Potentially increasing rates of hypertension in women of childbearing age and during pregnancy – be prepared!

**DOI:** 10.5830/CVJA-2010-074

**Published:** 2011-11

**Authors:** J Moodley

**Affiliations:** Women’s Health and HIV Research Unit, Department of Obstetrics and Gynaecology, Nelson R Mandela School of Medicine, University of KwaZulu-Natal, Durban, South Africa

**Keywords:** pregnancy hypertension, childbearing years, antihypertensive medication

## Abstract

**Abstract:**

The incidence of hypertension in young women is likely to increase in the near future because of the rising rates of the metabolic syndrome, obesity and dyslipidaemia worldwide. Consequently, more women will be on antihypertensive agents, which have the potential for teratogenecity. It is also likely that the increasing number of young women with essential hypertension who become pregnant will develop pregnancy-specific disorders such as pre-eclampsia. Health professionals should be aware of the effects of hypertension in women during the childbearing years, as well as the impact of pre-eclampsia on cardiovascular disease in later life. Pre-conception counselling skills, and knowledge on the use of antihypertensives and the changes that occur during pregnancy should be added to the clinical armamentarium of all health professionals.

## Abstract

Hypertensive disorders are the commonest medical complications occurring in pregnancy. They occur in approximately 6–8% of all pregnancies in the USA and cover a spectrum of disorders, such as chronic hypertension, gestational hypertension and pre-eclampsia/eclampsia syndrome.^1^ In South Africa, rates of hypertensive disorders in pregnancy are higher. A community-based study found a 12% incidence of hypertensive disorders in pregnancy in KwaZulu-Natal,^2^ while a tertiary facility-based study reported a rate of prevalence of 18%.^3^

Of recent concern is the increasing prevalence worldwide of obesity and the metabolic syndrome. Pregnant women who develop pre-eclampsia *de novo* share many of the risk features of the metabolic syndrome, namely, dyslipidaemia, obesity and insulin insensitivity. Therefore, increasing numbers of women could develop hypertension in their childbearing years and during pregnancy.

An increase in the numbers of young women presenting with hypertension would create challenges for general medical practitioners, obstetricians and specialist physicians. Firstly, significant hypertension requires investigation for an underlying cause. Secondly, the selection of antihypertensive agents for the treatment of essential hypertension in women of childbearing age poses challenges, as most antihypertensive medications are potentially teratogenic. Thirdly, several well-defined clinical hypertensive conditions, such as pre-eclampsia, are associated with high rates of maternal and neonatal morbidity and mortality. Lastly, hypertensive pregnancy disorders were traditionally not considered to have any long-term deleterious effects on cardiovascular health. However, recent studies have shown that pregnancy-specific hypertension is a risk factor for cardiovascular health later in life.^4^,^5^

Intensive counselling on the long-term impact of hypertensive disorders in pregnancy, the potential teratogenic effects of anti-hypertensive agents, appropriate diagnosis of pregnancy-specific hypertensive conditions and timely interventions therefore require an interdisciplinary approach if complications arising from these conditions are to be minimised.

## Treatment of essential hypertension in women of childbearing age

Although the Joint National Committee (JNC7) definition of hypertension and the treatment goals do not vary according to age and gender, the use of antihypertensive drugs in women of childbearing age and during pregnancy should be carefully considered in respect of their teratogenic potential.^6^ It is well established that angiotensin converting enzymes and receptor blockers have similar foetal effects in that they are associated with foetal renal agenesis, especially if used in the first trimester. However, several other antihypertensive agents seem to carry minimal teratogenic risks to the foetus (Table 1).

**Table 1.** Antihypertensive Drugs For Use During Pregnancy

*Drug*	*Route*	*Dose*	*Time*	*Action*	*Side effects*
Methyldopa	po	0.25–1.5 g twice/day	3–5 days	False neurotransmitter	Orthostasis, sleepiness, depression
Labetalol	po	200–1200 mg/d two or three times/day in divided doses	2–4 h acts within	Non-selective β-blockade	Tremulousness, headache
	iv	20–40 mg iv every 30 min as needed	5 min		
Nifedipine	po	30–120 mg/day	30 min	Calcium channel blocker	Oedema, orthostasis, dizziness
Monohydralazine	po	50–300 mg/d two or three times/day	1–2 h/20–30 min	Direct vasodilator	Lupus-like syndrome with chronic use
Dihydralazine	iv	10 mg every 2 h as needed			
	po	12.5–25 mg daily			
Hydrochlorothiazide	po	12.5–25 mg daily	3–5 d	Diuretic	
Emergency medications					
Labetalol as noted	iv				
hydralazine as noted	iv				
Nifedipine as noted	po				
Diazoxide	iv	30–50 mg every 5–15 min	2–4 min	Direct vasodilator	Hypotension, hypoglycaemia
Nitroprusside	iv	0.25 mg/kg/min	1–2 min	Direct vasodilator	Hypotension, cyanide toxicity if used > 4 h

po = per os; iv = intravenous

Women of childbearing age with class I hypertension usually do not require antihypertensive mediations.^6^ Successful lifestyle modifications and exercise in this group have been reported to demonstrate better blood pressure control.^7^-^10^ Furthermore, essential hypertension is independently associated with pre-eclampsia, and antihypertensive therapy in this group does not prevent the development of pre-eclampsia/eclampsia.

## Normal haemodynamic changes in pregnancy

Physiological changes in pregnancy may mimic signs of early congestive cardiac failure, and all health professionals should be aware of this. Briefly, changes in the cardiovascular system begin early in pregnancy, reaching a maximum at 28 weeks’ gestation. Within the first 12 weeks of pregnancy, the total intravascular plasma volume increases by 30–40%.^11^ Red blood cell mass increases by approximately 20%, but with the increased volume there is a relative decrease in the haematocrit.

**Fig. 1.**
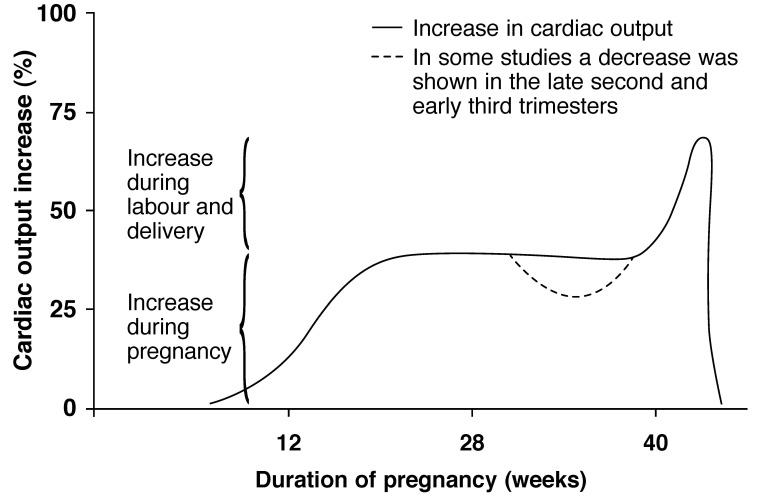
Maternal cardiac output during pregnancy.

The cardiac output increases on average by approximately 35%, commencing early in the first trimester, reaching a peak at 14 to 16 weeks and remaining at a plateau until labour. In labour, cardiac output increases moderately with each contraction and more appreciably with each expulsive effort in the second stage of labour. Most of the increase in cardiac output falls dramatically very soon after delivery (Fig. 1).

The increase in cardiac output in pregnancy is the result of an increase in pulse rate and stroke volume. The heart rate increases on average by 15 to 20 beats per minute and the stroke volume by 5–10 ml.^11^

Cardiac output is also influenced by maternal position. In the supine position (the patient lying on her back), venous return is reduced owing to pressure exerted by the pregnant uterus on the inferior vena cava. This reduced return leads to reduced output and hypotension (supine hypotension syndrome). This phenomenon is most often seen in late pregnancy.

## Arterial blood pressure (Fig. 2)

**Fig. 2.**
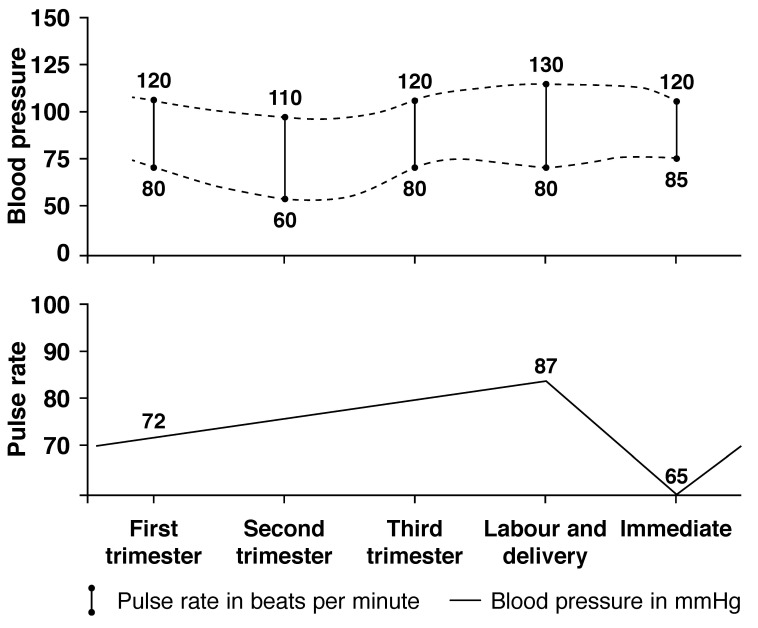
Variation in arterial blood pressure during pregnancy

In the lateral recumbent position, the blood pressure is higher in the upper arm than the lower (10–12 mmHg). While sitting, the blood pressure is slightly higher than in the supine position. Peripheral vascular resistance decreases during pregnancy due to the relaxing effect of progesterone on the smooth muscles. The subsequent decrease in blood pressure reaches a nadir in the second trimester compared with the early third trimester – the well-known drop in blood pressure.

The average decrease in systolic blood pressure is 5–10 mmHg and the decrease in diastolic is 10–15 mmHg. If this decrease fails to occur, it is reported that such women are more likely to develop hypertension in the third trimester of pregnancy.^12^

## Definition of hypertension in pregnancy

Hypertension in pregnancy is defined as systolic blood pressure ≥ 140 mmHg and/or diastolic blood pressure ≥ 90 mmHg (Korotkoff 5). It should be noted that because elevations of both systolic and diastolic blood pressure have been associated with adverse maternal and foetal outcomes, both are important. Also, detecting a rise in blood pressure from ‘booking’ or pre-conception blood pressure (> 30/15 mmHg) should lead to closer monitoring, but it is not diagnostic of hypertension in pregnancy.^12^

## Chronic hypertension

Chronic hypertension presents prior to pregnancy or before the twentieth week of gestation. It is reported to complicate 3% of all pregnancies and is more common in women who are obese or those over the age of 35 years. It is important to note that 20–30% of women with chronic hypertension go on to develop superimposed pre-eclampsia.^13^

## Pre-eclampsia/eclampsia syndrome

Pre-eclampsia is a syndrome of new-onset hypertension (> 140/90 mmHg) occurring after the twentieth week of gestation, with proteinuria (2+ on dipstick on two occasions six hours apart or > 3 g/24-hour urine collection).^12^

The aetiology remains elusive but current views suggest that it is a two-stage disorder.^14^ Put simply, the first stage is one of placental hypoperfusion, resulting in the release of a variety of substances (apoptotic cells, trophoblastic debris and anti-angiogenic factors) which cause multisystemic endothelial damage. The second stage presents as the clinical syndrome of hypertension, proteinuria, hepatic and central nervous system dysfunction.^14^ It is difficult to predict which organ system will be predominantly affected, but in general terms, the clinical signs of hypertension and proteinuria are the commonest. Pre-eclampsia therefore represents a spectrum of endothelial damage leading to downstream health effects.

Pre-eclampsia is divided into mild and severe categories. Severe disease is characterised by hypertension, namely, blood pressure values above 160/100 mmHg, proteinuria above 5 g per 24 hours, neurological symptoms (headache, visual disturbances), renal compromise (elevated serum creatinine and urea), hepatic dysfunction and haemolysis, and intra-uterine growth restriction. The presence of these symptoms and signs constitutes a medical/obstetric emergency, requiring admission to hospital and a multi-disciplinary approach to management.^12^

Although the exact aetiological mechanism is not known, epidemiological evidence suggests that pre-eclampsia affects the future health of the woman and her baby. Women with a history of pre-eclampsia are twice as likely to develop hypertension and two to five times more likely to have an ischaemic stroke in later life.^4^,^5^ It is unlikely that placental dysfunction on its own (stage I) leads to the pre-eclamptic disorder, but interactions with maternal constitutional factors (genetic, behavioural or environmental) may also be involved in the second stage of the disease process.

The ultimate therapy for pre-eclampsia is delivery of the baby, because the exact cause of the disease is not known. Clinical management is therefore individualised. In women with early-onset superimposed pre-eclampsia, blood pressure levels may increase quickly, be labile and require therapy as for a hypertensive emergency.^15^,^16^ In such circumstances, rapid lowering of high blood pressure and delivery of the baby, even if premature, may be required to prevent maternal complications.^17^ Treatment of high blood pressure alone will not prevent obstetric complications in such settings, and delivery of the foetus may be necessary to prevent adverse events in pregnancy, labour and the puerperium.

There is no doubt in the literature that women with sustained blood pressure values above 160 mmHg systolic and/or 110 mmHg diastolic should be treated with antihypertensive agents.^16^-^18^ On the other hand, there is little evidence to support antihypertensive therapy in pregnant women with blood pressure values below 160/100 mmHg. Nevertheless, in the clinical situation, there is a tendency to use antihypertensive medications in such circumstances, together with lifestyle modifications (diet and exercise).

Lifestyle modifications should ideally be initiated prior to conception in women with chronic hypertension, and continued in pregnancy. Exercise has been associated with reductions in gestational hypertension and a lower risk of eclampsia/pre-eclampsia. Due consideration however, needs to be given when making recommendations to maintain calorie intake and preventing injury.^8^-^10^

## Antihypertensive drugs in pregnancy

Table 1 lists the commonly used antihypertensive drugs. First-line agents include methyldopa, nifedipine and labetalol. Methyldopa is the most commonly used antihypertensive medication and the most studied. It has a long history of safety, is well tolerated and efficacious, and is often the first medication attempted in pregnant women. Methyldopa can be used three times daily, particularly if high doses are required. This dose makes it a costeffective method of treatment. Labetalol has also been studied extensively and found to be effective, although some studies have associated it with foetal growth restriction.

Angiotensin converting enzymes/angiotensin receptor blockers should be avoided in pregnancy and in women intending to become pregnant. These agents are associated with renal agenesis and foetal death.^19^ If a woman becomes pregnant while on angiotensin converting enzymes/angiotensin receptor blockers, these agents should be stopped immediately and alternate agents that have been found to be safe in pregnancy should be used. It is also important to note that if these agents are to be considered for use in young women of childbearing age, careful counselling and contraceptive advice must be offered.^13^,^20^

There are theoretical concerns regarding the use of diuretics during pregnancy. These include decreased placental perfusion and neonatal thrombocytopenia; therefore diuretics are not first-line agents. Calcium channel blockers are used in pregnancy. Most of the literature is on the use of nifedipine and it is regarded as safe for use in pregnancy.^18^,^21^ Other calcium channel blockers are probably safe although the manufacturers do not recommend their use. Selective b-blockers are considered safe during pregnancy but high doses are associated with neonatal hypoglycaemia and low birth-weight babies.^18^

Antihypertensive medication needs to be continued after delivery because blood pressure remains elevated for at least three to five days following delivery.^18^,^19^ Observational studies suggest that up to 25% of women with severe pre-eclampsia have ongoing postnatal hypertension.^22^ Consequently, a step-down approach to reducing the use of antihypertensive agents should be taken rather than stopping abruptly. Most antihypertensive agents are expressed in breast milk in minimal quantities.

## Hypertension in young women: pregnancy and the general practitioner

In South Africa, the general practitioner is often faced with women requesting a diagnostic test for pregnancy. It is incumbent on these professionals to ensure that blood pressure measurements are taken, so that careful counselling is given about the options of antihypertensive agents in respect of their safety in pregnancy. General practitioners also need to be aware of the supine hypotensive syndrome associated with pregnancy and the fact that Koratokoff 5 is used for measurement of diastolic blood pressure in pregnancy.^23^

Furthermore, general practitioners may be faced with a pregnant women presenting with severe hypertension during pregnancy, with or without symptoms and signs of a hypertensive emergency. These situations must be recognised and antihypertensive therapy initiated prior to referral to an appropriate health facility or specialist.

**Fig. 3.**
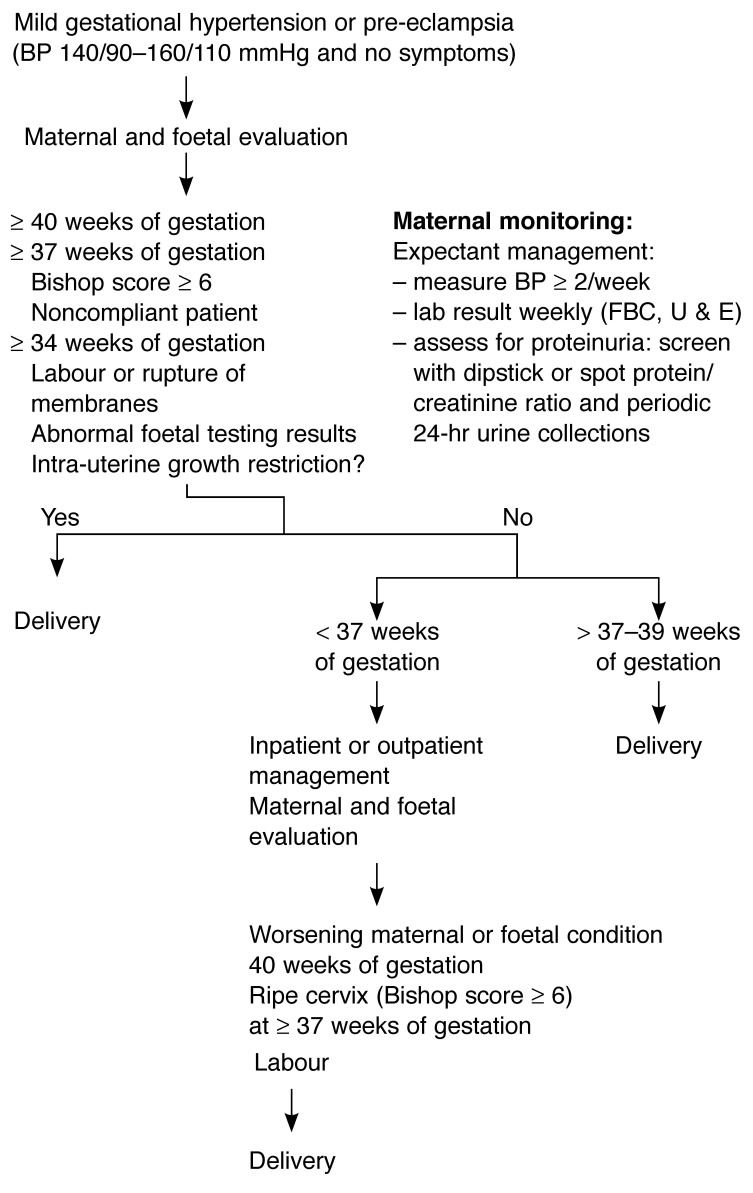
Management of mild gestational hypertension or pre-eclampsia.

**Fig. 4.**
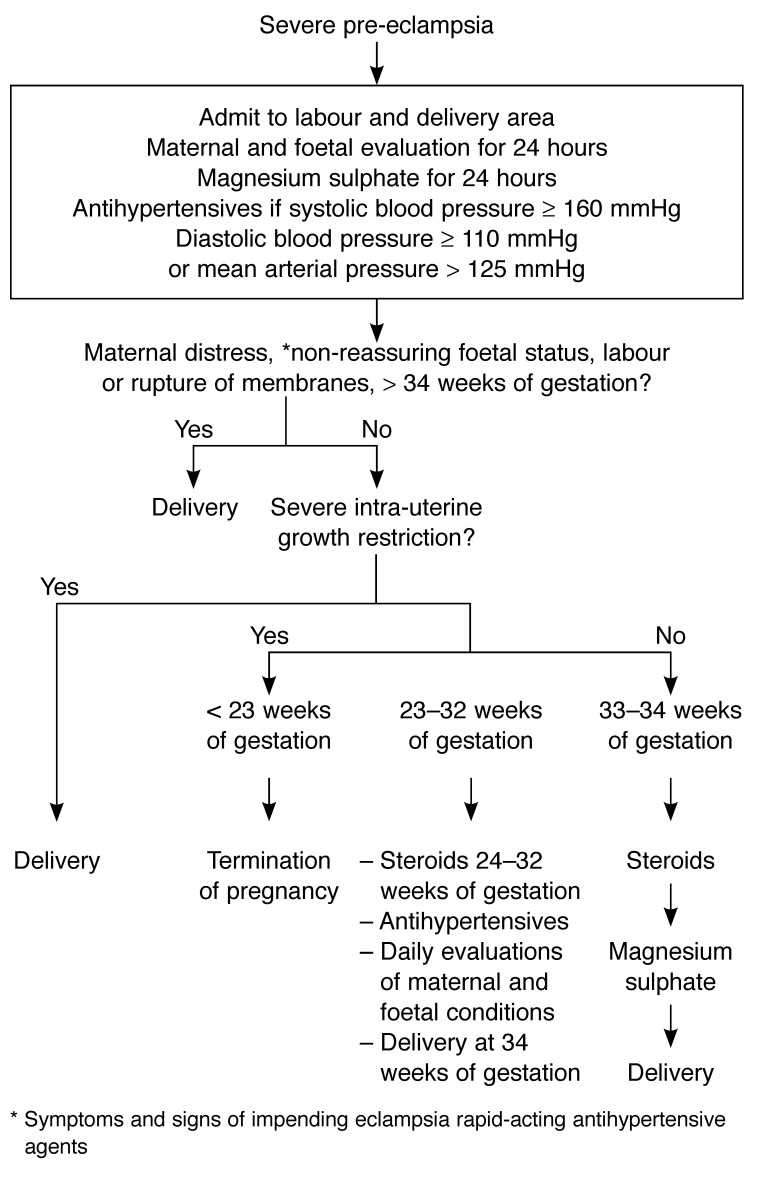
Management of severe pre-eclampsia.

Figs 3 and 4 summarise clinical management and may be useful for general practitioners, obstetricians and physicians. Ideally, such patients should be managed in referral centres, staffed by experts in hypertensive disorders of pregnancy.

## Conclusions

Hypertension in pregnancy is associated with significant maternal and perinatal morbidity and mortality. Regular blood pressure monitoring, detection of signs of pregnancy-associated hypertensive conditions and management by health professionals experienced in this field will minimise sequaelae associated with hypertensive disorders in pregnancy, and may have a positive impact on women’s cardiovascular events and outcomes years after the affected pregnancies.
